# Towards a Non-Invasive Technique for Healing Assessment of Internally Fixated Femur

**DOI:** 10.3390/s19040857

**Published:** 2019-02-19

**Authors:** Wing Kong Chiu, Benjamin Steven Vien, Matthias Russ, Mark Fitzgerald

**Affiliations:** 1 Department of Mechanical & Aerospace Engineering, Monash University, Wellington Rd, Clayton, VIC 3800, Australia; wing.kong.chiu@monash.edu; 2 The Alfred Hospital, 55 Commercial Road, Melbourne, VIC 3004, Australia; M.Russ@alfred.org.au (M.R.); M.Fitzgerald@alfred.org.au (M.F.); 3 National Trauma Research Institute, 89 Commercial Road, Melbourne, VIC 3004, Australia

**Keywords:** internal fixation, fractured femur, healing assessment, dynamic response, spectral analysis

## Abstract

The lack of a quantitative method to adequately assess fractured bone healing that has undergone fixation limits prognostic capabilities on patients’ optimal return to work. This paper addresses the use of vibrational analysis to monitor the state of healing of a plate-screw fixated femur and supplement the current clinical radiographic assessment. This experimental study involves an osteotomised composite femur specimen enclosed by modelling clay to simulate the damping effect of overlying soft tissues. Epoxy adhesives are applied to the fractured region and to simulate the healing process. With the instrumentation described, the cross-spectrum and coherence are obtained and analysed in the frequency domain over a period of time. The results suggest that it is crucial to analyse the cross-spectrum and proposed healing index to quantitatively assess the stages of healing. The results also show that the mass loading effect due to modelling clay did not influence the proposed healing assessment technique. The findings indicate a potential non-intrusive technique to evaluate the healing of fractured femur by utilising the vibrational responses.

## 1. Introduction

This paper is an extended version of the conference paper, Wing Kong Chiu, Benjamin Steven Vien, Matthias Russ and Mark Fitzgerald, Healing assessment of an internally fixated femur using vibration analysis, 7th Asia-Pacific Workshop on Structural Health Monitoring 2018, Hong Kong, 12 November 2018.

**Figure 1:**
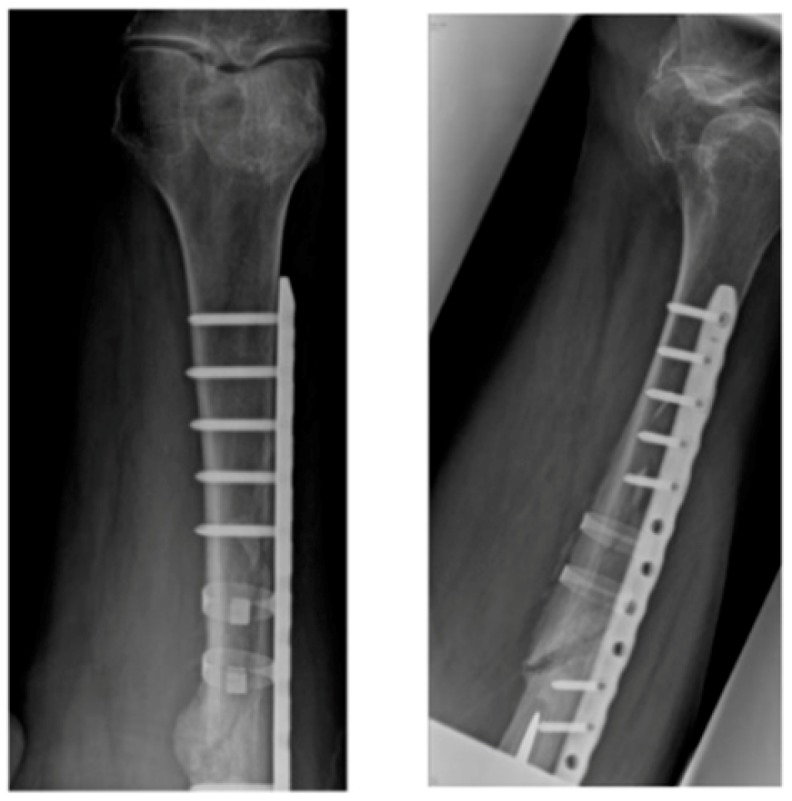
Internal plate-screw fixation.

Internal fixations are a common standard treatment for a fractured femur to correct alignment, provide mechanical stability, allow weight bearing, and prompt early use of the limb while the bone is healing. In comparison to external fixation, internal fixation has been associated with decreased pin tract sepsis and joint stiffness [1]. The plate-screw fixation healing efficiency involves reducing the fracture gap such that bone contact develops at the fracture interface, immobilising the fracture site [2]. Internal fixation allows patients to return to normal function earlier than casts and splints would normally allow, as well as reduces the incidence of non-union and mal-union. Figure 1 shows examples of the installed plate and screws internal fixation on a long bone.

However, the difference in modulus between the plate and bone and the induced compressive stress by over-tightened screws disturb the bone vascularity and bone resorption underneath the plate, thus compromising healing, strength, and longevity [2]. Furthermore, the bone stress-shielding due to the stiff internal fixations adversely delays the bone healing. There has been a considerable amount of research on plate-screw fixation to improve fracture healing and prevent stress-shielding and bone loss [3,4,5,6,7]. An essential part of the treatment is accurately determining the healing progression and unification of the fixated fractured femur. The healing process of a fractured bone is complicated, and delayed union, mal-union and non-union are common occurrences due to the delicate balance between the anabolic and catabolic phases of normal healing [8]. Prior to allowing the patient to return to their normal activities, the degree of healing is often assessed through clinical interpretation of images from X-rays or CT scans. These radiography techniques are known to provide qualitative assessment, of which its accuracy of interpretation highly depends on the physician’s experience [9,10,11,12,13,14]. Therefore, it is desirable to develop a novel, analytical technique to objectively and accurately determine the state of healing. 

There are a variety of measurement techniques available for evaluating fracture healing, including ultrasound, direct static measurement, and vibration measurement to measure the stiffness of an internally fixated fractured bone [9,15,16,17,18,19,20,21,22,23,24,25,26,27,28,29,30,31]. Shah et al. [16] reported on clinical measurements on 11 patients for whom they assessed the change in stiffness of fractured tibial shaft as it healed. In their study, the fractured tibia was placed in a cast, which was removed during measurement and replaced afterwards. As the fractured tibia healed, they measured significant changes in its stiffness of the tibia, which was measured statically using a Goniometer and a load cell. Claes et al. [17] also substantiated the use of stiffness to assess the state of healing of 100 tibial fractures. Borchani et al. [18] reported the use of an instrumented fixation device to monitor bone healing by integrating measurement sensors within the internal fixation. Nemchand [19] investigated wireless telemetered instrumented intramedullary nail implants and reported sufficient sensitivity to monitor strain with callus growth of Young’s Modulus 0.2 MPa. However, their recent instrumentations have yet to produce a reliable set of significant data for potential clinical use. 

The existing literature commonly reports on strain readings used to determine the extent of healing [18,19,20,21,22]. However, others have reported that there is no correlation between strain and bone healing [21]. Talaia et al. study [20] used fibre Bragg gratings to assess the stiffness of callus formation of fractured bone by loading intact and plate femur to 600N. However, at this particular loading, it may result in further damage to a fractured femur.

There is interest in using dynamic vibrational analysis techniques for quantitative healing assessment [9,23,24,25,26,27,28,29,30,31]. Cornelissen et al. [24] utilised vibrational measurements on a healing tibia, which revealed bending modes and frequency shifts during the healing process. Unfortunately, they were unable to track the frequency shift due to errors which may have been caused by mode coupling. Sekiguchi et al. [25] investigated 41 in vivo cases of tibial fractures by inducing vibration to the tibia with a customised hammer at the medial malleolus and a piezoelectric receiver at the medial region of the tibial tuberosity. Their study reported that a completely healed long bone has higher frequency waveforms compared to a newly fractured bone. An in vivo study by Tower et al. [26] examined a vibrational response up to 400 Hz of a fractured tibia with an external fixator and internal fixation which included intramedullary nails, unreamed and reamed nails, plates and inter-fragmentary fixation. The Tibial Stiffness Index, which is the ratio of the resonant frequency of the healing tibia and the intact tibia on the other leg, was then formulated to potentially quantify the healing. Despite a good correlation with the traditional methods, the authors did not recommend this method for clinical use due to a significant error amount in the index. 

More recently, extensive works by Ong et al. [29,30] reported the possibility to assess the healing of a fractured femur with external fixation. Ong et al. demonstrated that the state of healing of a femur could be quantified by using PVDF film sensing elements on the external fixation pin and an impulse excitation on the external fixation. Furthermore, the effect of mass loading from soft tissue on the healing assessment was also investigated and discussed in the Ong et al. study [30]. Tsuchikane [32] reported that muscles were the most significant damper. The soft tissues, joints, and fibula increase the ‘apparent’ weight and dampen tibial vibration, a recognized obstacle in analysing the vibrational response. Additionally, Chiu et al. [31] concluded that the vibrational response of the femur treated with a plate fixation is not significantly influenced by transverse or oblique fracture orientation. Nakatasuchi et al. [27] simulated the healing process by injecting epoxy adhesive into the fracture and observed a steady increase in bending mode frequency during healing. These studies suggest that the dynamic vibration response could be used for quantitative healing assessment.

Zhang et al. [33] demonstrated the mass of an accelerometer severely dampened the dynamic response of a light-weight cantilever bar. Previous studies using the dynamic response to account for stiffness changes as an indication of the state of healing relied on the definition of the frequency response function of the femur. Furthermore, these experiments require consistent forcing and knowledge of the input force measured with an instrumented impactor [26,29,30,34]. 

The paper will present a methodical approach, investigating assessment of the state of healing by measuring the dynamic response of a plate-screw fixated femur. The two-sensor development method is demonstrated to measure the dynamic stiffness of the fractured femur treated with a plate-screw fixation, to mitigate the necessity for an instrumented impactor and to facilitate the transition to clinical use with minimal complexity. The change in the stiffness was used to define the state of healing of the plate-screw fixated femur. A composite bone model test specimen was used, and the healing was simulated by using long cure time epoxy adhesives applied to the fractured region. Additionally, this investigation also incorporates the mass loading effects of soft tissue, which is simulated by highly damped modelling clay around the fixated femur. Chiu et al. [31] serves a reference on the type of excitation that should be considered to distinguish the key responses of the construct as a function of healing. This study is an extension of our previous works on utilising structural health monitoring techniques for bone healing assessments [29,30,31,35,36]. The findings demonstrate the ability to quantify the state of healing in the presence of the simulated soft tissues by defining the required spectral response that is associated with bone healing. Furthermore, the outcome of this project proceeded to the recently developed healing device (Australia Patent: 2019900018) [37] by Chiu, Russ and Fitzgerald, and clinical trials of the device are currently under preparation.

## 2. Experimental Methods

**Figure 2:**
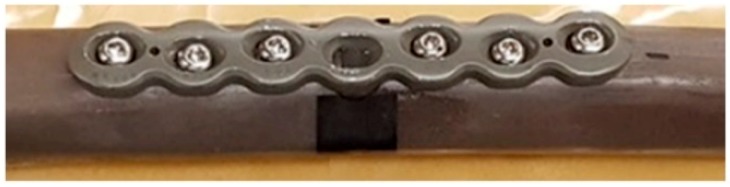
Close up view of the plate-screw fixation.

**Figure 3:**
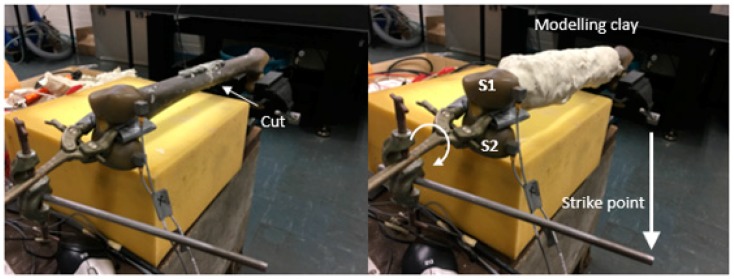
Experiment test setup indicating saw blade cut, modelling clay, two accelerometers and strike point.

The experimental specimen is Sawbones^®^ composite femur fixated with Stryker^®^ (Kalamazoo, MI, USA) plate-screw fixation, which was created from an AxSOS small fragment locking plate system, shown in Figure 2. In many efficacy trials, authors have employed the fiberglass-reinforced epoxy Sawbones^®^ composite bone due to its similar mechanical characteristics to an average human cadaver bone [38,39,40,41]. The proximal (upper) femur head was fastened with a vice rigidly attached to a heavy block concrete and securely gripped with a set of 3D-printed vice clamps matched to the femur head geometry. Two B&K Type 4507 unidirectional accelerometers were attached to the distal (lower) femur head and orientated to measure the acceleration in the y-axis direction (perpendicular to the long bone). The acquired signals from the accelerometers were processed by a 2-channel B&K Photon+. Plasticine, modelling clay, is chosen to fundamentally and functionally represent mass loading effects of soft tissue because of its density and damping, and it can be moulded with maximal contact (no slippage). Figure 3 shows a schematic of the experimental rig and the application of the input loading. 

Previous works by Ong et al. [29,30] used an instrumented impact force hammer to acquire results in an orderly procedure. In this study, the input force is intentionally not recorded, and the forcing location is not fixed. This test set-up allowed force variability during the experiment and facilitated the evaluation of the robustness of the measurement technique proposed. The 2-sensor system measures the dynamic vibrational responses of the fixated femur as it heals with a statistical evaluation to validate the acquired data. 

A saw blade was used to perform a mid-shaft osteotomy of the composite femur, and a plate-screw fixation was then installed. Prior to the experiment, tapes were placed over the fracture site to form a mould to ensure uniform filling of adhesive epoxy, which is consistent with previous work on simulating healing across fracture site [42]. A 30-min cure time two-part adhesive epoxy was used to simulate the healing process. Prior to the epoxy filling, modelling clay was partially added to the femur. Once the fracture site was filled with epoxy, the plate-screw fixation is installed and then the remaining modelling clay is applied to enclose the femur, as shown in Figure 3. It should be noted that upon mixing the two-part epoxy, it initiates the hardening process thus the epoxy cure while the test specimen is being prepared. The mass of the modelling clay used was approximately 1 kg. The final mass of the composite femur, plate-screw fixation, and modelling clay is shown in Table 1.

The initial recording of time t = 0 s denotes the first set of experimental results collected immediately after fully enclosing the femur with modelling clay. The experiments were conducted at regular intervals as the epoxy cured–simulating ‘*healing*’, in the osteotomized region of the femur. In order to span the entire curing process, data was recorded until 180 min at 2-min intervals for the first 100 min and 5-min intervals for the remaining 80 min. The frequency bandwidth of 10 kHz with a frequency resolution of 1.56 Hz and the spectra were averaged over 10 samples to obtain a good signal-to-noise ratio. It should be noted that the spectrum was observed to stabilise after an average of 7 samples. The measurement at each healing stage took approximately 30 s to perform, which was considered to be insignificant compared to the epoxy curing time. The cross-spectrum between the two accelerometers, coherence function, and phase were recorded in the frequency domain. The sensitivity frequency peaks and shifts with high coherence in the spectral analysis throughout the curing process are determined in each case. In spectral analysis, the relation between the measured two signals is shown in the coherence function. A high coherence value indicates the data acquired are associated with the changes in the stiffness resulting from the simulated healing of the fixated femur. Furthermore, in the 2-sensor setup, the phase function assists in identifying the mode at the associated frequency peaks. On completion of the experiment, the test specimen was re-used and the test procedure repeated. A total of three experiments were conducted. 

## 3. Results

### 3.1. No Mass Loading Effect on Healed Plate-Screw Fixated Femur

**Figure 4:**
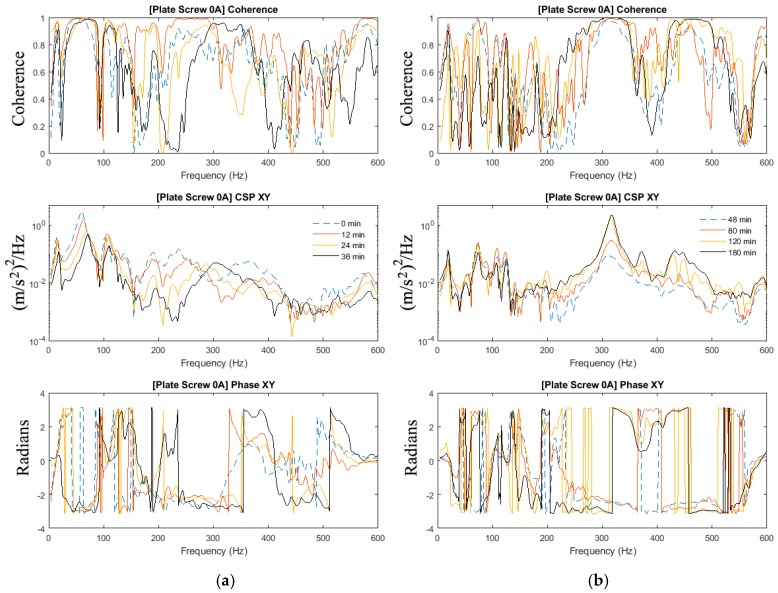
Coherence, cross-spectra and phase functions of Test #0A experimental investigations; (**a**) from 0 to 36 min and (**b**) from 48 to 180 min.

**Figure 5:**
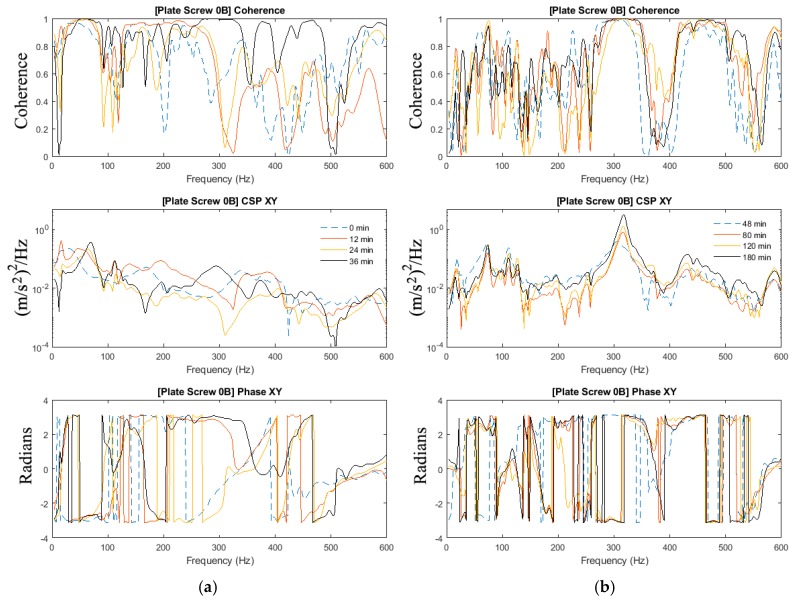
Coherence, cross-spectra and phase functions of Test #0B experimental investigations; (**a**) from 0 to 36 min and (**b**) from 48 to 180 min.

The results presented did not require the acquisition of the force input. The cross-spectra calculated from the 2-sensor measurement methodology is able to indicate the state of healing of the fractured femur. In this respect, the coherence function presented is required to confirm that the development of the cross-spectra is attributed to the change in the state of healing and not due to extraneous noise. The magnitude and phase of the cross-spectrum and coherence function of the plate-screw fixated femur without the inclusion of modelling clay are shown in Figure 4 and Figure 5. There are fundamentally two key observations that can be made from these results:

1. The frequency peaks at 15 Hz; 74 Hz; 111 Hz and 127 Hz are observed to increase as the fractured region “healed”. This is attributed to the stiffness increase due to the simulated healing.
2. The effects of healing on the dynamic response of the fixated long bone is better shown in the vicinity of 315 Hz and 450 Hz. It is evident that the dynamic response is only evident towards the later part of the experiment when the fractured femur has “healed”.

The manner in which the internal fixation is applied to the femur accounts for the dynamic stiffness observed in the frequency bandwidth up to 127 Hz. It is noted that the coherence at these frequencies is close to unity even at the commencement of the experiment. As the healing is allowed to progress, the increase in stiffness is manifested in the shift of these frequency peaks. However, in the vicinity of 315 and 450 Hz, the coherence is observed to approach unity as “healing” progresses. The development of the cross-spectra in these bands is a testament to the state of healing of the fixated femur.

### 3.2. Mass Loading Effect on Healed Plate-Screw Fixated Femur

Whilst the findings of the bare composite femur are relevant to healing assessment, the effects of mass loading on the femur due to the presence of soft tissue must be addressed. Indeed, Tsuchikane [32] reported that the soft tissues act to increase the “apparent” weight of long bones and as a result will dampen the vibration response. To determine the effects of mass-loading on the healing assessment methodology presented above, we need to first investigate how the response of the cross-spectra to the presence of added-mass (modelling clay) on the unfractured fixated femur. 

**Figure 6:**
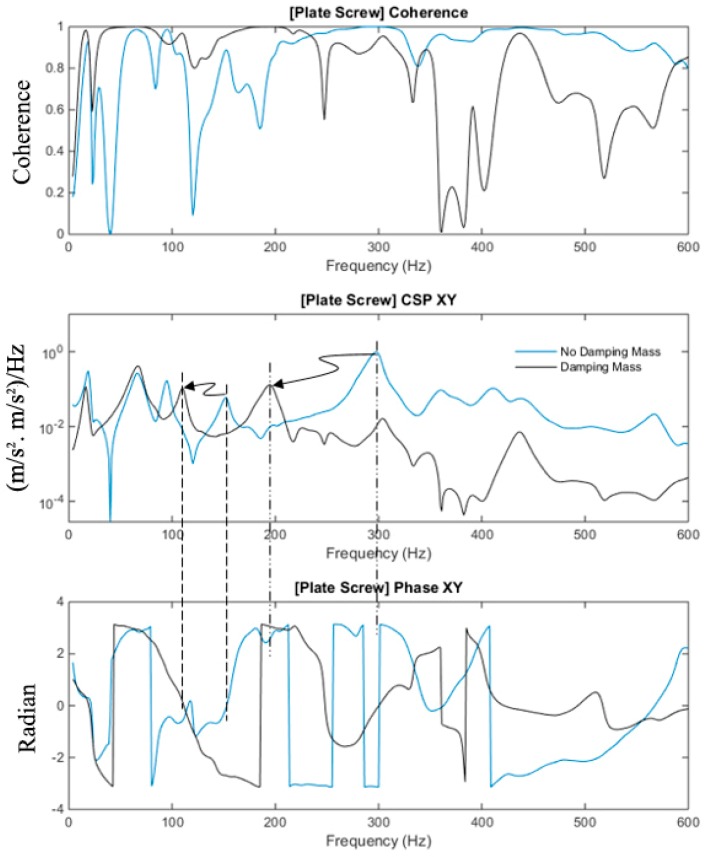
Coherence, cross-spectra and phase with and without damping mass of the fully healed plate-screw fixation.

The magnitude and phase of the cross-spectrum and coherence function of the plate-screw fixated femur with and without the modelling clay are shown in Figure 6. The response below 100 Hz is not affected by the mass loading of the modelling clay, refer to Figure 6. However, at higher frequencies, the modelling clay mass contribution is significantly evident.

The first out-of-phase modes of the construct affected by mass loading, i.e., with and without mass loadings, were observed at approximately 195 Hz and 300 Hz, respectively. The first in-phase modes affected by mass loading, i.e., with and without mass loadings, were measured at approximately 105 Hz and 160 Hz, respectively. These modes are associated with the fixated femur as indicated by its significant coherence values. Furthermore, the effect of the modelling clay dampens the magnitudes of the cross-spectral as shown in Figure 6. The modelling clay is seen to damp out the higher modes, and this damping effect concurs with previous findings [33]. The combined effects of spectra compression and damping are evident and, therefore, it is important to consider these effects when determining the veracity of the healing assessment methodology.

### 3.3. Healing Assessment of the Fixated Femur with Mass Loading

**Figure 7:**
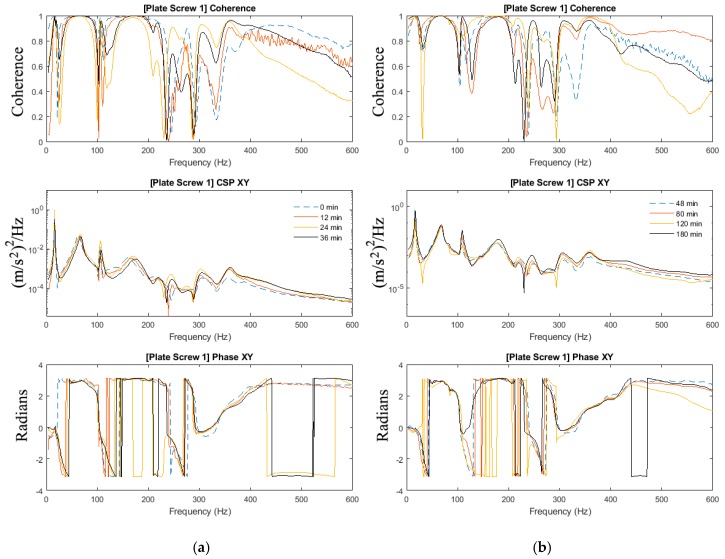
Coherence, cross-spectra and phase functions of Test #1 experimental investigations; (**a**) from 0 to 36 min and (**b**) from 48 to 180 min.

**Figure 8:**
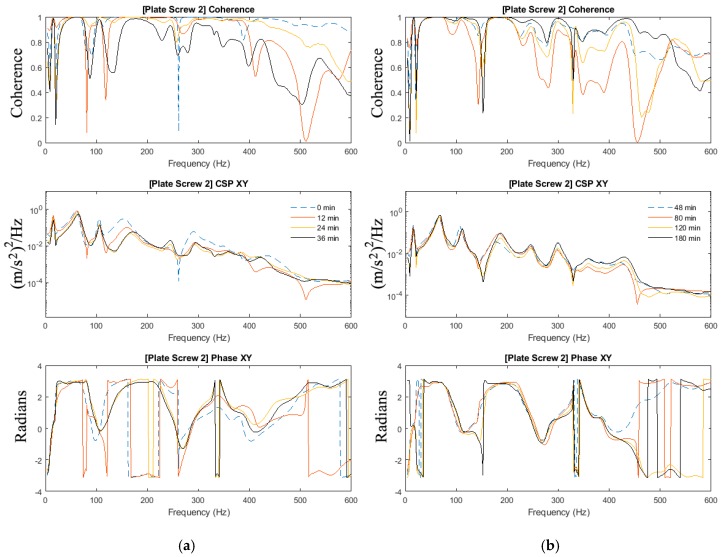
Coherence, cross-spectra and phase functions of Test #2 experimental investigations; (**a**) from 0 to 36 min and (**b**) from 48 to 180 min.

**Figure 9:**
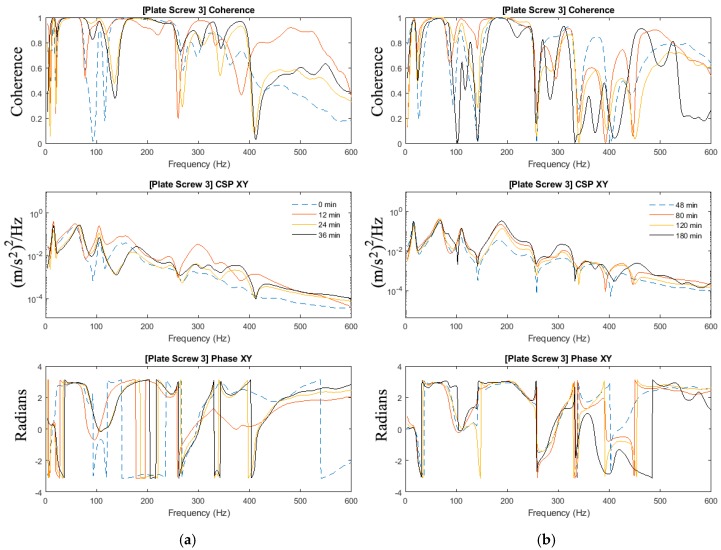
Coherence, cross-spectra and phase functions of Test #3 experimental investigations; (**a**) from 0 to 36 min and (**b**) from 48 to 180 min.

In Section 3.2, the effects of added-mass are apparent and thus highlighting the importance to investigate the mass-loading effect on the ability to determine the state of healing in the mass-loaded fixated fractured femur. The experiments in Section 3.1 of this paper are repeated with the addition of the modelling clay to simulate mass loading arising from soft tissues. Figure 7, Figure 8 and Figure 9 show the magnitude and phase of the cross-spectrum and the corresponding various stages of healing.

The key observation and characteristic of the results are as follows:

1. At frequencies below 200 Hz, in contrast to the case without mass loading, it is observed that frequency peaks at 16 Hz; 67 Hz and 109 Hz are not sensitive to the state of healing. The response in the vicinity of 179 Hz, however, was affected by the state of healing. The frequency associated with the peak response at this frequency was noted to increase as healing progressed that corresponded to the increased stiffness due to the simulated healing. It was also noted that at frequencies below 200 Hz, the dynamic responses of the fully healed specimens were identical in all three cases presented.
2. For the frequencies above 200 Hz, where the effect of mass loading was noted to be significant, the three tests conducted show that the state of healing is reflected by the frequency response measured. The increasing magnitude of the cross-spectra and the corresponding value of the coherence attest healing progression. This observation is similar to the case without mass loading.

As observed in Section 3.2, the effects of mass-loading on the spectra response are distinct. Similarly in Section 3.1, given that the forcing input is not acquired, the coherence function serves to verify the significance of the spectra peaks measured. It is observed that:

1. Compared with the results in Figure 4 and Figure 5 (no mass loading), the effects of the change in stiffness due to healing on the spectrum are severely hampered by the presence of the modelling clay.
2. In spite of the presence of mass-loading, the spectra development in the frequency band above 200 Hz, whilst significantly damped, is still evident.

Therefore, a definition of the state of healing of a mass-loaded femur needs to be conducted with careful inspection of the cross-spectra along with the coherence function, where relying on the changes in the frequency response alone is not sufficient. Both the effects of the healing on the natural modes of the fixated femur and the effects of the healing on the overall mass loading of the fixated femur have to be taken into consideration when assessing its state of healing. 

### 3.4. Healing Index for Quantitative Evaluation

**Figure 10:**
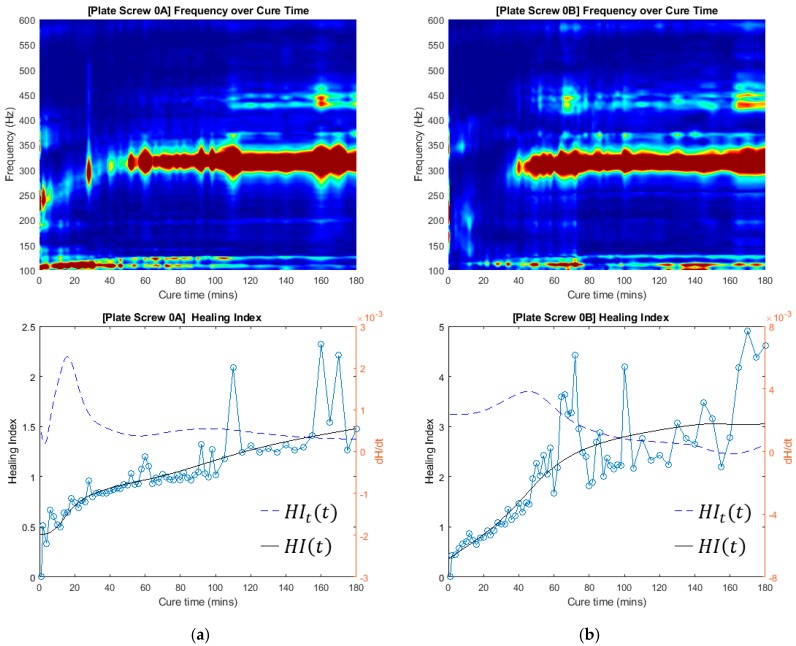
Frequency over cure time and Healing Index of two experimental investigations with no mass damping. (**a**) Test #0A; (**b**) Test #0B.

**Figure 11:**
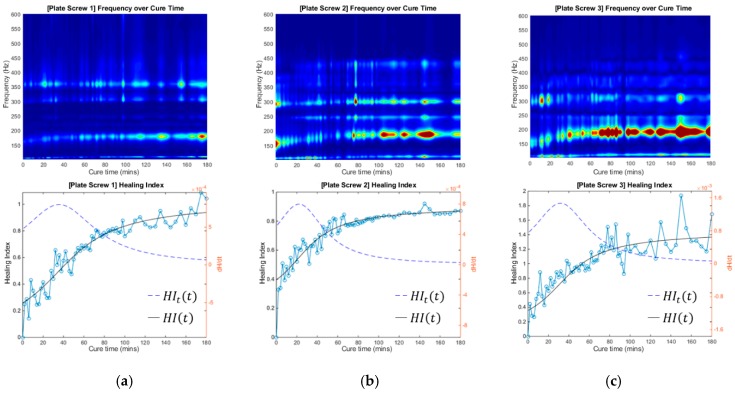
Frequency over cure time and Healing Index of three experimental investigations with mass damping. (**a**) Test #1; (**b**) Test #2; (**c**) Test #3.

The cross-spectrum for the three tests were processed by using the normalised healing index, as defined by (1). The frequency bandwidth of interest is between 0 Hz and 600 Hz. The healing index is normalised to the cross-spectrum obtained from the first experiment conducted; initial healing index (i.e., time zero, see Figure 10 and Figure 11). The gradient of the healing index defined by (2) is also included in these results. These results show the behaviour of the healing index and provide means to analyse and deduce the state of healing of the fractured femur.

(1) $$HI\left(t\right)=\frac{1}{Initial\;healing\;index}\overset{600}{\underset{0}{\int }}\left|CS\left(f,t=i\right)-CS\left(f,t=0\right)\right|df$$

where, $\;\;Initial\;healing\;index\;=\overset{600}{\underset{0}{\int }}CS\left(f,t=0\right)df$

(2) $$HI_{t}\left(t\right)=\frac{d}{dt}\left(HI\left(t\right)\right)$$

The healing index and the time-derivative of the normalized healing index (*HI_t_*) served to identify stages of healing, as calculated by (2). Whilst the healing index curve can be seen to asymptote as healing progresses, a definitive statement on the state of healing is best made in conjunction with the cross-spectrum and the rate of change of the healing index simultaneously to identify and evaluate the healing process. 

The evolution of the cross-spectra with respect to the healing of the femur with and without modelling clay is presented in Figure 10 and Figure 11. The cross-spectrum PSDs measured during the experiment are presented in this intensity plot. Figure 10 and Figure 11 show the application of the proposed healing index to the sets of experimental results. It is observed that the significant changes in the vicinity of 315 Hz and 179 Hz contribute to healing without and with the mass damping, respectively. Furthermore, this indicated that the healed femur healing index is at least 80% higher than the fracture fixated femur, even with the presence of mass loading. Furthermore, the healing index curve shows the progression of healing and asymptotes with increasing time, which is a significant improvement compared to previous work by Ong et al. [30]. The robustness of the manner in which the state of healing is determined is evident in Figure 10 and Figure 11. The measurement technique did not require knowledge of the input stimulus. The variability of the strike point will lead to experimental error, as shown in the healing index curves in Figure 10 and Figure 11. However, the curve fitted to the healing index, its associated gradient, and with the consideration of cross-spectral provide a definitive statement on the state of healing.

## 4. Discussion

Previous studies have employed epoxy adhesive in the means of simulating bone healing and osseointegration to represent similar curing process of changing in material properties and increasing stiffness in the femur. [27,43,44]. Physiologically, the fracture bone healing begins from blood clotting to callus ossification, which is similar to the process of viscous elastic epoxy from liquid to solid state [45,46,47]. However, the curing adhesive epoxy is not an exact representation of bone healing and the material properties when fully solidified are not equivalent to the parent material (composite bone). 

Some variations in the healing index such as the point of inflection and index values are observed. Given the fact that the adhesive epoxy hardens immediately after mixing the two components and the volume portions are not controlled, this gives rise to variation in cure duration and adhesive quality. Furthermore, the preparation time to assemble the specimen, application of the adhesive and installation of the modelling clay are also different in each specimen. Nevertheless, the healing index is shown to be sensitivity to the curing of the adhesive. It is also noted that the effect of highly damped modelling clay did not affect this method ability to assess the healing progress. 

The findings serve as a basis for further study on using the fracture femur vibrational response for quantitatively healing assessment and continuous development of the healing device. In a practical setting, the proposed measurement technique is incorporated into an extra corporeal device, which is secured externally on the distal part of the femur [37]. Indeed, the necessity for clinical trials is essential for validating this methodology, determining secondary influences in operating and environmental conditions and optimising prototype design to incorporate this 2-sensor setup, and to further progress with the investigation.

## 5. Conclusions

This paper reports assessment of healing progression of a simulated fractured femur treated with plate-screw fixation using dynamic vibrational responses. The 2-sensor deployment methodology was presented in this study to mitigate the common use of an instrumented impactor for healing assessments using vibrational analysis. The effect of the modelling clay as a simulated soft tissue influenced the dynamic response of the fixated femur. However, this mass loading effect is minimal on the proposed healing assessment technique. The findings emphasized the necessity for the proposed healing index, the rate of change of the healing index and the associated cross-spectrum to be concurrently analysed to evaluate the state of healing. This study demonstrated a non-intrusive method by utilising vibrational analysis to assess bone healing as a quantitative approach—which allows further insight to potentially predict and prevent common construct failures. Future works are currently being investigated on healing assessment for clinical use. 

## Figures and Tables

**Table 1:** Mass of composite femur specimen with plate-screw fixation.

Specimen	Mass
Sawbone composite femur	512 g
Sawbone composite femur + plate-screw fixation	598 g
Sawbone composite femur + plate-screw fixation + modelling clay	1598 g
